# Optimization of Pectin Extraction from Melon Peel as a New Source of Pectin and Pectin Hydrolysate with Prebiotic Potential

**DOI:** 10.3390/foods13162554

**Published:** 2024-08-16

**Authors:** Saroya Bilraheem, Sirasit Srinuanpan, Benjamas Cheirsilp, Apichat Upaichit, Fusako Kawai, Uschara Thumarat

**Affiliations:** 1 Environmental Biotechnology Laboratory, Faculty of Agro-Industry, Prince of Songkla University, Hat Yai, Songkhla 90110, Thailand; saroya.bl@hotmail.com; 2 Center of Excellence of Microbial Diversity and Sustainable Utilization, Faculty of Science, Chiang Mai University, Chiang Mai 50200, Thailand; sirasit.s@cmu.ac.th; 3 Center of Excellence in Innovative Biotechnology for Sustainable Utilization of Bioresources, Faculty of Agro-Industry, Prince of Songkla University, Hat Yai, Songkhla 90110, Thailand; benjamas.che@psu.ac.th (B.C.); apichat.u@psu.ac.th (A.U.); 4 Graduate School of Environmental and Life Sciences, Okayama University, 1-1-1 Thshimanaka, Kita-ku, Okayama 700-8530, Japan; fkawai@okayama-u.ac.jp

**Keywords:** fruit wastes, melon peel, pectin extraction, pectin hydrolysate, prebiotic activity score

## Abstract

Food wastes have a large number of functional ingredients that have potential for valorization. Melon peels are increasingly produced as waste in food industries in Thailand. This study aimed to optimize pectin extraction conditions from melon peel for its prebiotic potential. Optimization was conducted using a response surface methodology and Box–Behnken experimental design. An analysis of variance indicated a significant interaction between the extraction conditions on extraction yield and degree of esterification (DE). These include pH and solvent-to-sample ratio. The conditions for the extraction of pectin with low DE (LDP), medium DE (MDP) and high DE (HDP) were optimized. Pectin hydrolysate from LDP, MDP and HDP was prepared by enzymatic hydrolysis into LPEH, MPEH and HPEH, respectively. LDP, MDP, HDP, LPEH, MPEH and HPEH were compared for their efficiency in terms of the growth of three probiotic strains, namely *Lactobacillus plantarum* TISTR 877, *Lactobacillus casei* TISTR 390 and *Enterococcus faecium* TISTR 1027. Among the samples tested, HPEH showed the highest ability as a carbon source to promote the growth and prebiotic activity score for these three probiotic strains. This study suggests that melon peel waste from agro-industry can be a novel source for prebiotic production.

## 1. Introduction

Currently, people’s concerns on health and quality of life are increasing. The demand for food ingredients from natural products with functional and health properties has increased. Prebiotics are famous items due to their benefits on the promotion of human gut microbiota [1]. Prebiotics include dietary fibers that humans cannot digest and absorb in the digestive tract, although they can be degraded by gut the microflora [2]. Prebiotics include fructo-oligosaccharides, isomalto-oligosaccharides, lactilol, lactosucrose, lactulose and inulin. Pectin and pectin-derived oligosacharides (POSs) are a new source of prebiotics since they have been reported for their prebiotic properties and improve the growth of potential probiotics such as *Lactobacillus* spp. *Bifidobacterium faecali, Bacterium prausnitzii* and *Roseburia* spp. [3,4,5,6]. Several studies have demonstrated diverse effects, such as apoptosis in human colonic adenocarcinoma cells [7,8], their potential ability to protect heart health, mitigation of heavy metal-induced damage, anti-obesity impacts, antibacterial traits, and antioxidant properties [9].

The pectin extracted from fruit peel biomass displays potential as a promising source for producing pectic oligosaccharides or pectin hydrolysates. These forms demonstrate capabilities in aiding probiotic growth and presenting prebiotic potential [1,2,3]. Pectinase enzymes can hydrolyze pectin into monosaccharides or oligosaccharides, which can be used as carbon sources for probiotics [2]. In a previous study by Chatterjee et al. [10], pectin was extracted separately from the peels of various fruits, including orange, tomato, banana, guava and watermelon, to evaluate the prebiotic properties of the pectin derived from each fruit. The findings showed that tomato peel-derived pectin exhibited the highest growth stimulation for *Lactobacillus casei* [10]. POSs from mango peel were evaluated for their prebiotic effects on *Lactobacillus reuteri* DSM 17938 and *Bifidobacterium animalis* TISTR 2195. The results demonstrated valuable scores of prebiotic activities for both *B. animalis* TISTR 2195 (7.76) and *L. reuteri* DSM 17938 (6.87) [11]. Considering the degree of esterification (DE) of pectin between low-methoxyl pectin (LMP; DE < 50%) and high-methoxyl pectin (HMP; DE ≥ 50%), Olano-Martin et al. [12] reported that LMP has a higher prebiotic index (PI) than HMP. An evaluation of the prebiotic potential of POSs with a low or high DE of pectin from orange peel found that the PIs of low-DE POSs and high-DE POSs were 0.12 and 0.08, respectively [12].

The utilization of agricultural byproducts and wastes as sources of prebiotics is a useful way to valorize and eliminate waste. Therefore, many attempts have been made with various byproducts and wastes to comply with the government policies on zero-waste production. However, none of those attempts have investigated the prebiotic properties of pectin and pectic oligosaccharide components from melon peel. The production of melon in Thailand is popular nowadays. The average annual yield was 984,100 kg in 2018 [13]. During fruit processing, melon peels constitute approximately 20–30% of the total fresh weight, serving as byproducts that are abundant in pectin and dietary fiber [14,15]. According to Hartati and Subekti [16], melon peels are composed of approximately 20% cellulose, 23% hemicellulose, 10% lignin and 13% pectin. The extraction process for pectin involves various factors such as pH, temperature, time and solid-to-solvent ratio (SSR), which influence extract quality parameters like extraction yield and properties including methoxyl content (MeO), DE, and anhydro-galacturonic acid content (AUA), resulting in differences in prebiotic potentials between pectin and pectin hydrolysate [12,17]. Therefore, it is imperative to optimize the extraction variables and evaluate the prebiotic potentials of pectin and pectin hydrolysate.

This study aimed to optimize pectin extraction from melon peel for the production of pectin and pectin hydrolysate with prebiotic potential. This research introduces an approach to enhancing the value of byproducts sourced from Thai-fruit industrial wastes and suggests a framework conducive to sustainable industrial development.

## 2. Materials and Methods

### 2.1. Preparation of Melon Peel

The commercial Hami melon cultivars (*Cucumis melo* var. *saccharinus*) utilized in this research were obtained from a nearby fruit market. To ensure homogeneity, all samples were collected in the same week of the year. After collection, approximately 10 kg of the fruit peels was removed, cleaned, and dried in an oven at 60 °C for 48 h. Then, the dried peels were ground into powder by grinder and sieved through a 40-mesh sieve. The processed powder was stored in a dry environment before being used for experimental analyses [18].

### 2.2. Extraction of Pectin from Melon Peel

The pectin extraction from melon peel was conducted in accordance with the methodology described by Khan et al. [19]. Pectin was extracted under varied conditions: temperatures of 75, 85, and 95 °C, extraction times of 60, 120, and 180 min, pH levels at 1.0, 2.0, and 3.0, and different solid-to-solvent ratios (SSR) of 10, 30, and 50 *v*/*w*. Following the filtration process with two layers of gauze, the pectin extract underwent centrifugation at 8000× *g* for 15 min to remove residual particles. Subsequently, the resulting supernatant was precipitated using a 95% ethanol solution (at a ratio of 1:2% *v*/*v*) for 24 h at 4 °C. The polysaccharides underwent two ethanol washes. Finally, the resultant precipitated pectin was vacuum-dried at 60 °C until reaching a constant weight. The extraction yield was then determined using the following formula:

(1) $$\%\;\mathrm{Yield}\;=\frac{\mathrm{weight}\;\mathrm{of}\;\mathrm{dried}\;\mathrm{pectin}\;\mathrm{extract}\;(g)}{\mathrm{weight}\;\mathrm{of}\;\mathrm{dried}\;\mathrm{peel}\;\mathrm{powder}\left(g\right)}\;\times \;100$$

The determination of DE followed the titrimetric approach by Raji et al. [18]. Initially, 0.2 g of dried pectin extract was amalgamated with ethanol and then dissolved in 20 mL of deionized water at 40 °C. Following the thorough dissolution of the pectin extract, five drops of phenolphthalein were introduced into the solution. After dissolving the pectin extract, five drops of phenolphthalein were added. Following this, the solution was subjected to titration utilizing 0.5 M NaOH, and the volume of NaOH solution causing the color change was recorded as the initial titer (v_1_). Subsequently, 10 mL of 0.5 M NaOH was added, and intense agitation of the solution for 15 min was initiated; then 10 mL of 0.5 M HCl was introduced, and the solution was stirred until the pink color disappeared. Titration with 0.5 M NaOH was performed, and the volume used was recorded as the saponification titer or final titer (v_2_). DE was calculated based on the following formula:

(2) $$\%\;\mathrm{DE}\;=\frac{v2}{v1+v2}\times \;100$$

### 2.3. Optimization of the Pectin Extraction Conditions of Melon Peel Using the Response Surface Methodology (RSM)

The RSM was used to anticipate the optimal pectin extraction conditions for melon peel, which were yield and DE. The optimization process was carried out following Raji et al. [18] and Prakash Maran et.al. [20] with a slight modification. Twenty-nine hydrolysis trials were randomly run per Box–Behnken (BBD) experimental design with independent variables, including temperature (X_1_: 75, 85, 95 °C), extraction time (X_2_: 60, 120, 180 min), pH (X_3_: 1, 2, 3) and SSR (X_4_: 10, 30, 50 mL/g) employed at three equally spaced tiers (−1, 0, +1). The conditions for the extraction of pectin with a low (LDP), medium (MDP) and high (HDP) degree of esterification were optimized. Pectin hydrolysate from LDP, MDP and HDP was subjected to enzymatic hydrolysis; the hydrolysates from LDP, MDP and HDP were LPEH, MPEH and HPEH, respectively.

### 2.4. Properties of Pectin Extract from Melon Peel

Pectin from the models (LDP, MDP and HDP) was characterized by some properties such as equivalent weight (Eq.wt), MeO, DE⁠ and AUA using a modification designed by Mohamed [21].

### 2.5. Prebiotic Properties of Extracted Pectin and Pectin Hydrolysate (PEH)

#### 2.5.1. Preparation of Pectin and Pectin Hydrolysate from Melon Peel

Pectin samples from melon peel extraction in optimized conditions with three levels of DE, low (DE 40–50%), medium (DE 60–70%) and high (DE 80–90%), were used to study prebiotic potentials. PEH was prepared through the hydrolysis of pectin with pectinase. The pectin extract at 2% *w*/*v* was used to react with commercial pectinase (30,000 U, Reach biotechnology, Thailand) at pH 4 and 45 °C for 6 h in an incubator shaker. Then, the reaction was stopped by boiling for 10 min. The hydrolyzed biomass was centrifuged at 5000× *g* for 15 min. The three levels of PEH were named LPEH (PEH hydrolyzed from pectin with 40–50% DE), MPEH (PEH hydrolyzed from pectin with 60–70% DE) and HPEH (PEH hydrolyzed from pectin with 80–90% DE).

#### 2.5.2. Preparation of Probiotics

Three strains of probiotic microorganisms were used for testing the prebiotic properties: *L. plantarum* TISTR 877, *L. casei* TISTR 390 and *E. faecium* TISTR 1027. Microbial strains were cultured in MRS broth and incubated at 37 °C for 48 h. The amount of each microorganism after incubation at 10^6^ CFU/mL was used for further testing of the prebiotic properties of pectin and PEH.

#### 2.5.3. Effect of the DE of Pectin and PEH on the Growth of Probiotics

Probiotic cultures, comprising 0.1 mL with 10^6^ CFU/mL, were added to 10 mL of MRS broth containing 2% pectin or 2% PEH and incubated at 37 °C for 0, 12, 24 and 48 h. After inoculation in the test medium, the final amount was 10^4^ CFU/mL. Then, serial dilution and pour plate on MRS agar were conducted to count viable cells as CFU/mL. The controls included a positive control (with glucose) and a negative control (without glucose).

#### 2.5.4. Evaluation of Prebiotic Activity Score (PAS)

The assessment of prebiotic activity was conducted following a method previously reported [22]. *L. plantarum* TISTR 877, *L. casei* TISTR 390 and *E. faecium* TISTR 1027 were represented as probiotic cultures (P), while *Escherichia coli* DMST 4212 was chosen to represent the enteric species (E). The experiment was conducted by measuring the number of CFU/mL prior (P^0^ and E^0^) and subsequent (P^24^ and E^24^) to incubation for 24 h at 37 °C on 2% (*w*/*v*) glucose (P_G_ and E_G_), with 2% (*w*/*v*) of the test pectin or pectin hydrolysate as the prebiotic (P_x_ and E_x_) as reported in [23]. The experiment was performed three times for each assay. Subsequently, the prebiotic activity score was determined using Equation (3):

(3) $$\mathrm{Prebiotic}\;\mathrm{activity}\;\mathrm{score}\;(\mathrm{PAS})=[({LogP}_{x}^{24}-{LogP}_{x}^{0})/({LogP}_{G}^{24}-{logP}_{G}^{0})]-[({LogE}_{x}^{24}-{LogE}_{x}^{0})/({LogE}_{G}^{24}-{logE}_{G}^{0})].$$

### 2.6. Statistical Analysis

The optimization of the pectin extraction conditions was performed using RSM Design-Expert 7.0 software (Stat-Ease 2005). Each analysis was represented by mean values (±SD). An ANOVA analysis through Minitab 14.0 was conducted to determine potential significant differences among the treatments.

## 3. Results

### 3.1. Optimization of Pectin Extraction from Melon Peel

#### 3.1.1. Model Fitting and Statistical Analysis

The RSM was applied to enhance the conditions for the extraction of pectin from melon peel. Data from 29 experimental trials conducted using central composite design (CCD) were obtained, involving four distinct independent factors: temperature (X1), extraction time (X2), pH (X3) and solvent-to-sample ratio (X4). In this study, the summary model indicated the importance of a two-factor interaction model, offering valuable insights for pectin yield and DE. The predicted model for pectin yield and DE was a quadratic model. The pectin yield of melon peel obtained from 29 experimental trials ranged from 2.77 to 22.44%, which was similar to the pectin yield obtained from orange peels by Marin et al. [24] but higher than that from the yield of passion fruit peel (2.25–14.60%) [25]. Khan et al. [26], Hashmi et al. [27] and Dehankar et al. [28] have reported the highest pectin yields of 20%, 20.12% and 21%, respectively. Meanwhile, the DE of extracted pectin ranged from 41.96 to 91.80%, which was broader than the extraction of passion fruit peel (41.67–67.31%) [25]. The quantity, quality and characteristics of pectin differ depending on plant origins, extraction techniques and parameters. [29]. The following are the second-order equations associated with the extraction yield and DE:

pectin yield (%) = 20.49460 − 1.04076X_1_ + 0.09640X_2_ + 23.98358X_3_ + 0.65615X_4_ + 0.00714X_1_^2^ − 0.000355833 X_2_^2^ − 4.58975X_3_^2^ − 0.00638688X_4_^2^ + 0.0003625X_1_X_2_ − 0.083750X_1_X_3_ + 0.0019625X_1_X_4_ − 0.015542X_2_X_3_ − 0.0000645833X_2_X_4_ − 0.13375X_3_X_4_ (4)

de (%) = 149.86462 − 2.23924X_1_ + 0.26224X_2_ − 48.02965X_3_ − 0.29087X_4_ + 0.018179X_1_^2^ + 0.000640764X_2_^2^ + 17.03438X_3_^2^ + 0.00867173X_4_^2^ − 0.0043934X_1_X_2_ − 0.022362X_1_X_3_ − 0.00445979X_1_X_4_ − 0.010865X_2_X_3_ − 0.000684182X_2_X_4_ + 0.15893X_3_X_4_ (5)

where X_i_ represents the coded independent factor (X_1_ = temperature, X_2_ = extraction time, X_3_ = pH, X_4_= solvent-to-sample ratio).

In our investigation, an analysis of variance (ANOVA) was employed to validate the significance of the models, as shown in Table 1. Significantly low *p*-values for both yield and DE (less than 0.001) and an insignificant lack of fit (higher than 0.05) indicate the suitability of the model in representing the responses for these parameters [30]. On the other hand, the adequacy of the models was examined by evaluating the determination coefficients for yield and DE [31,32] with the value of the determination coefficient (95.70% and 98.44%, respectively), the adjusted determination coefficient (91.40% and 96.88%, respectively) and the predicted determination coefficient (76.50% and 92.21%, respectively). The significant R2 and Adj-R2 values attest to the high level of precision and reliability of the conducted experiments [33].

#### 3.1.2. The Relationship between Factor Variables and Extraction Yield

**Figure 1:**
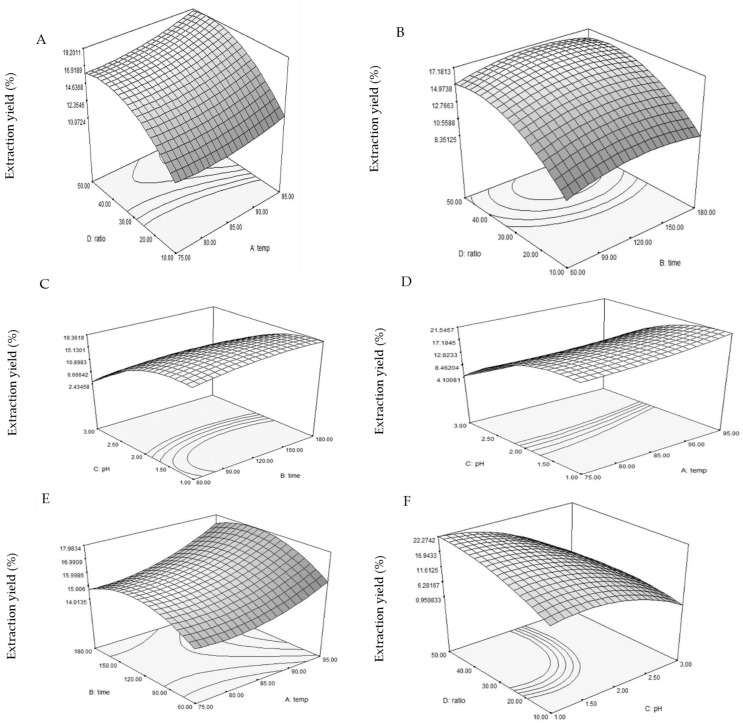
Three-dimensional response surface representations illustrating the impact of temperature (°C), extraction time (min), pH and the solvent-to-solid ratio (ml/g) on the extraction yield (%). The interactions are depicted in subfigures: (**A**) solvent-to-solid ratio vs. temperature; (**B**) solvent-to-solid ratio vs. extraction time; (**C**) pH vs. extraction time; (**D**) pH vs. temperature; (**E**) extraction time vs. temperature; and (**F**) solvent-to-solid ratio vs. pH.

The experiments were conducted over different pH levels (1–3), and the outcomes are depicted in Figure 1C,D,F. The results notably demonstrate an increase in the extraction yield of pectin as the pH levels decrease. The acidic solvent at a lower pH effectively solubilized insoluble pectin, converting it into a soluble form and consequently enhancing the extraction yield of pectin [18]. It should also be noted that at high pH levels, pectin accumulates, preventing its release and thereby lowering the extraction yield of pectin [34]. The results of this study align with research on extracting pectin from apple pomace, sugar beet pulp, pumpkin and pomegranate peel [35,36,37,38]. Temperature is considered one of the critical factors influencing pectin extraction yield. The findings demonstrate an increase in pectin yield as temperature rises (Figure 1A,D,E). Increased temperature enhances solvent solubility and penetration into plant tissue, leading to an increased pectin extraction yield [39]. This outcome was consistent with pectin extraction from peach pomace [40] and melon peel [18]. Regarding the impact of extraction time, longer extraction times boosted pectin yield (Figure 1B,C,E). This observation aligned with those previously reported by Chen et al. [41] and Zheng et al. [42]. Also, a high value of the solvent-to-sample ratio enhances pectin yield from melon peel extraction (Figure 1A,B,F), possibly due to the enlarged contact surface area between the melon peels and the solvent [33].

#### 3.1.3. The Relationship between Factor Variables and DE

**Figure 2:**
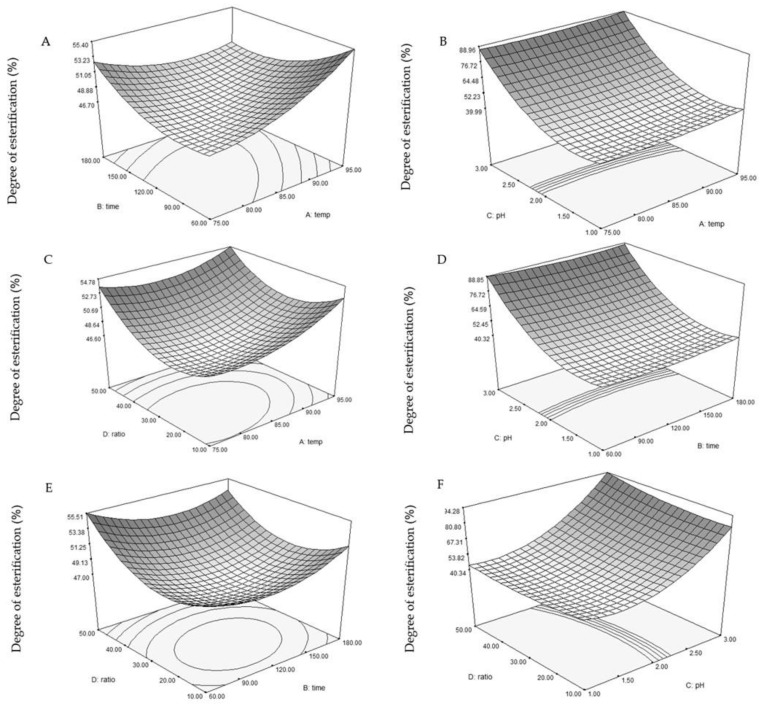
Three-dimensional response surface representations illustrating the impact of temperature (°C), extraction time (min), pH, and solvent-to-solid ratio (mL/g) on the extraction DE (%). The interactions are depicted in subfigures: (**A**) extraction time vs. temperature; (**B**) pH vs. temperature; (**C**) solvent-to-solid ratio vs. temperature; (**D**) pH vs. extraction time; (**E**) solvent-to-solid ratio vs. extraction time; and (**F**) solvent-to-solid ratio vs. pH.

An increase in the DE was observed as pH and the SSR were increased while extraction time and temperature were decreased, as illustrated in Figure 2. This occurrence is attributed to polygalacturonic chain de-esterification occurring under harsh conditions such as high temperature, extended duration and low pH [43]. Similar patterns were observed in pectin extraction from dragon fruit peel by Tang et al. [44] and from melon peel by Raji et al. [18]. In addition, the values of DE reduced with a decrease in extraction temperature, a decrease in time, an increase in the SSR and a decrease in the pH value. The pH of the extraction solution and the SSR significantly influenced the DE, consistent with previous findings [45]. Significant interactive effects included a linear pH effect (*p* < 0.0001), a cubic pH effect (*p* < 0.0001), an SSR effect (*p* = 0.0096) and a quadratic pH-SSR effect (*p* = 0.0488) on the DE. Similar findings were reported by Li et al. [45].

### 3.2. Optimization Conditions of Factor Variables for the Yield and DE

To explore the impact of varying pectin esterification levels on prebiotic properties, the factor variables were optimized to maximize pectin yield at low, medium and high DE levels by solving Equations (4) and (5). The optimal conditions for the highest yield for the three levels of methylated pectin are shown in Table 2. Also, all of this methylated pectin was characterized in terms of equivalent weight (Eq.wt), methoxyl content (MeO) and anhydrouronic acid content (AUA) (Table 2). Based on the model, three conditions of pectin extraction that produced LDP, MDP and HDP were optimized to compare their prebiotic properties. The optimal extraction conditions for the LDP were identified as an 85 °C temperature, a 166 min duration, a pH of 1, and an SSR of 50 mL/g. The MDP displayed optimal conditions at an 85 °C temperature, a 120 min duration, a pH of 2.2, and an SSR of 50 mL/g, while the HDP exhibited ideal conditions at an 85 °C temperature, a 90 min duration, a pH of 2.7, and an SSR of 50 mL/g. The corresponding responses of the LDP, MDP and HDP were 46.17%, 56.65% and 88.38% DE, respectively. From Table 2, the extraction temperature of pectin was specified for a maximum of 85 °C to reduce the energy consumption required for extraction for model optimization. The optimal temperature for the effect of LDP, MDP and HDP extraction on pectin yield was the same at 85 °C, which is the maximum temperature condition. It was also demonstrated by Chan and Choo [17] and Vriesmann et al. [46] that increasing the temperature significantly enhanced the extraction of pectin from cocoa husks with the assistance of citric acid. The heated conditions facilitated the dissolution of pectin and other pectic substances enclosed in the cell wall (protopectin), thereby elevating the overall pectin yield. The optimal SSR for the effect of LDP, MDP and HDP extraction on pectin yield was the same at 50 mL/g, which is the maximum. Increasing the temperature and SSR showed an increase in pectin yield and %DE. The DE value decreased with increasing extraction time and decreasing extraction pH, consistent with a report by Li et al. [45], which emphasized the significant impact of pH and time on the DE of mango peel pectin, whereas temperature showed no considerable effect. To achieve both maximum yield and DE, the extraction conditions should involve shorter extraction periods with a high pH value. In the case that the highest yield but lowest DE were required, the conditions for extraction should be a higher extraction duration with a low pH. Since a lower extraction duration with a high pH were mild conditions, some of the methyl group still remained in the galacturonic acid residues. The yield of pectin under the conditions used to extract HDP was lower than the yield of pectin in the conditions of LDP extraction, but the purity of the HDP was better (78.43% of AUA) than that of the LDP (13.05% of AUA) when the temperature and SSR were the same. Finally, the three pectin types LDP, MDP and HDP were used to study prebiotic properties.

### 3.3. Prebiotic Properties of Extracted Pectin and PEH on Growth of Probiotics

**Figure 3:**
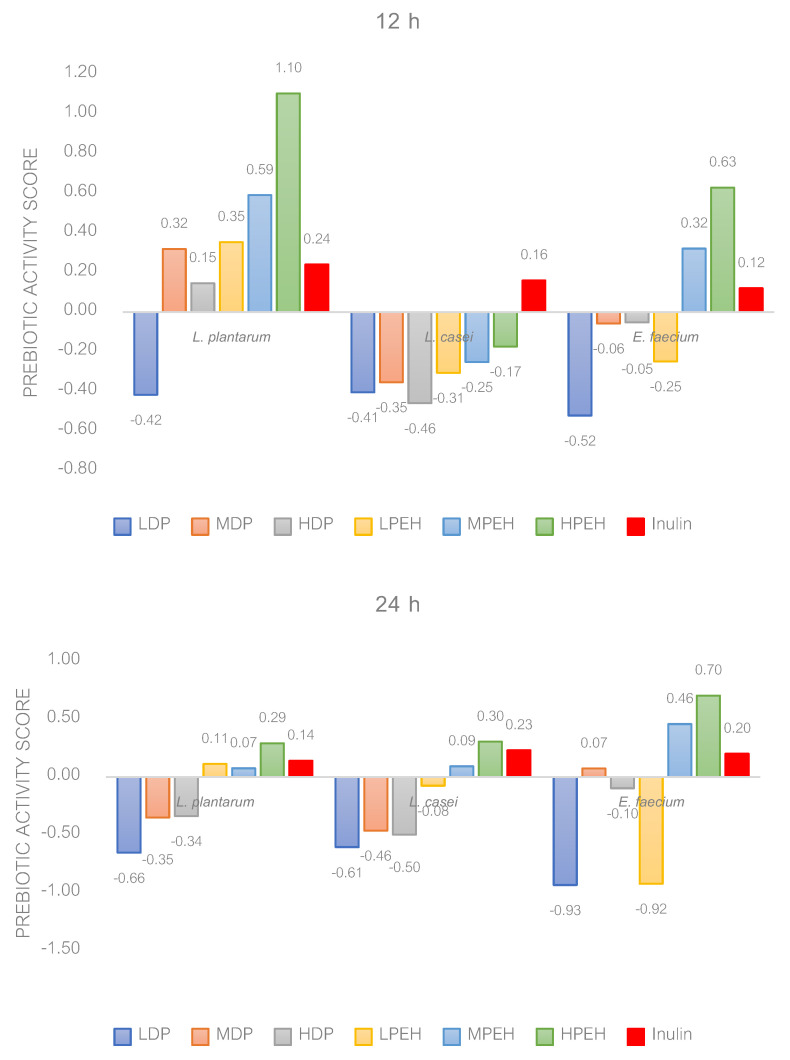
The prebiotic activity scores of bacterial cultures grown in MRS supplemented with 2% (*w*/*v*) pectin (LDP, MDP, and HDP) or pectin enzyme hydrolysates (PEHs: LPEH, MPEH, and HPEH), as well as inulin, are presented. Data are shown from three independent repeats.

The growth of three probiotic strains, namely *E. faecium* TISTR 1027, *L. plantarum* TISTR 877 and *L. casei* TISTR 390, in the glucose-free MRS media, containing three types of pectin (LDP, MDP and HDP) or PEHs (LPEH, MPEH and HPEH), is shown in Table 3. During the early stages of incubation, the growth of *L. casei* TISTR 390 was promoted by HDP, LPEH, MPEH and HPEH, but only pectin hydrolysate promoted its growth when the incubation time increased to 36 h, similar to the results for *E. faecium* TISTR 1027. In contrast, the growth of *L. plantarum* TISTR 877 was only promoted by PEHs at the beginning of incubation, but both pectin and pectin enzyme hydrolysate promoted their growth after 36 h of incubation time. The fact that the probiotic counts were lower in the media containing pectin at the beginning of incubation might result from the acidity following the pattern of LDP > MDP > HDP. The presence of HPEH in the culture media as a carbon source resulted in the highest growth for all three probiotic strains. Despite glucose in the MRS media displaying comparable growth, it is crucial to recognize that glucose is absorbable and provides calories. On the other hand, pectin oligosaccharides in HPEH cannot be absorbed by the body, offering no caloric value [2]. Figure 3 illustrates the prebiotic activity scores of pectin and pectin hydrolysates from melon peel for *L. casei* TISTR 390, *L. plantarum* TISTR 877 and *E. faecium* TISTR 1027, calculated from cfu/mL data after 12 and 24 h of incubation using Equation (3). *L. plantarum* TISTR 877 exhibited higher prebiotic activity when grown in MRS broth with added PEH compared to pectin, and the highest score was recorded for HPEH (1.10). After 12 h of fermentation, HPEH exhibited prebiotic scores 4.6-fold and 5.2-fold greater than inulin for *L. plantarum* TISTR 877 and *E. faecium* TISTR 1027, respectively. Furthermore, the prebiotic activity score for HPEH obtained from melon peel was higher than the prebiotic activity score of citrus-derived POSs for *L. paracasei* LPC-37 (0.41) and *Bifidobacterium bifidum* ATCC 29521 (0.92) [5]. The pectin enzyme hydrolysates demonstrate significant prebiotic potential, especially for *L. plantarum* TISTR 877, as pectinase is expected to break down pectin into galacturonic acid and pectic oligosaccharides [47].

Our results clearly showed that PEH from melon pectin offers growth-enhancing ability for the tested probiotics. The data support the prebiotic potential of POSs or pectin hydrolysate from citrus peel [2,5] and sugar beet pulp [9]. Thus, PEH is a highly beneficial component for food supplements, offering prebiotic properties.

## 4. Conclusions

In conclusion, both the yield and the degree of esterification of pectin were significantly affected by the pH value and SSR used in the extraction process. These conditions encompassed an extraction temperature of 85 °C, an SSR of 50 mL/g for 166 min and a pH of 1. Both pectin and pectin hydrolysate from melon peel have the potential to enhance the growth of *Lactobacillus plantarum* TISTR 877, *Lactobacillus casei* TISTR 390 and *Enterococcus faecium* TISTR 1027. The prebiotic activity score of HPEH was the highest. This is the first time the feasibility of a high value-added product such as prebiotics from melon peel has been shown in the field of utilizing fruit industry wastes.

## Figures and Tables

**Table 1:** The statistical analysis of variance using ANOVA evaluates the variability and significance within the chosen model for pectin yield and degree of esterification (DE).

Source	Sum of Squares	DF	Mean Square	F-Value	*p*-Value	Source	Sum of Squares	DF	Mean Square	F-Value	*p*-Value
(A) Yield						(B) DE					
Model	1015.57	14	72.54	22.26	<0.0001 *	Model	7673.79	14	548.13	63.15	<0.0001 *
X_1_	13.98	1	13.98	4.29	0.0573	X_1_	25.42	1	25.42	2.93	0.1091
X_2_	3.34	1	3.34	1.02	0.3286	X_2_	0.0046	1	0.0046	0.0005	0.9819
X_3_	652.10	1	652.10	200.09	<0.0001 *	X_3_	5635.77	1	5635.77	649.33	<0.0001 *
X_4_	129.89	1	129.89	39.86	<0.0001 *	X_4_	35.59	1	35.59	4.10	0.0624
X_1_^2^	3.31	1	3.31	1.01	0.3309	X_1_^2^	21.44	1	21.44	2.47	0.1384
X_2_^2^	10.64	1	10.64	3.27	0.0923	X_2_^2^	34.52	1	34.52	3.98	0.0660
X_3_^2^	136.64	1	136.64	41.93	<0.0001 *	X_3_^2^	1882.18	1	1882.18	216.86	<0.0001 *
X_4_^2^	42.34	1	42.34	12.99	0.0029 *	X_4_^2^	78.04	1	78.04	8.99	0.0096 *
X_1_ X_2_	0.19	1	0.19	0.058	0.8131	X_1_ X_2_	27.79	1	27.79	3.20	0.0952
X_1_ X_3_	2.81	1	2.81	0.86	0.3692	X_1_ X_3_	0.20	1	0.20	0.023	0.8815
X_1_ X_4_	0.62	1	0.62	0.19	0.6703	X_1_ X_4_	3.18	1	3.18	0.37	0.5545
X_2_ X_3_	3.48	1	3.48	1.07	0.3191	X_2_ X_3_	1.70	1	1.70	0.20	0.6648
X_2_ X_4_	0.024	1	0.024	0.0074	0.9328	X_2_ X_4_	2.70	1	2.70	0.31	0.5861
X_3_ X_4_	28.62	1	28.62	8.78	0.0103 *	X_3_ X_4_	40.42	1	40.42	4.66	0.0488 *
Residual	45.63	14	3.26			Residual	121.51	14	8.68		
Lack of Fit	42.43	10	4.24	5.32	0.0608	Lack of Fit	99.46	10	9.95	1.80	0.2992
Pure Error	3.19	4	0.80			Pure Error	22.05	4	5.51		
Total	1061.19	28				Total	7795.30	28			
Std. Dev.	1.81	R-Squared	0.9570			Std. Dev.	2.95	R-Squared	0.9844		
Mean	12.89	Adj R-Squared	0.9140			Mean	57.40	Adj R-Squared	0.9688		
C.V.	14.00	Pred R-Squared	0.7650			C.V.	5.13	Pred R-Squared	0.9221		
PRESS	249.40	Adeq Precision	16.424			PRESS	607.35	Adeq Precision	24.341		

* Significance level at 95%.

**Table 2:** Recommended solutions for optimal pectin extraction and their characteristics.

	Optimal Condition				Response				Characteristics		
					Predicted Value		Measure Value				
	Temp (°C)	Time (min)	pH	Ratio (mL/g)	Yield (%)	DE (%)	Yield (%)	DE (%)	Eq.wt (g)	MeO (%)	AUA (%)
LDP	85	166	1	50	22.83	45.20	21.42 ± 1.45	46.17 ± 0.71	210.55 ± 3.13	12.63 ± 0.55	13.05 ± 1.41
MDP	85	120	2.2	50	14.22	60.00	12.02 ± 2.74	56.65 ± 1.03	303.10 ± 6.49	13.37 ± 0.27	55.30 ± 12.90
HDP	85	90	2.7	50	7.28	80.00	6.53 ± 1.06	88.38 ± 0.71	2678.57 ± 252.54	8.84 ± 0.22	78.43 ± 5.48

LDP = pectin with low DE; MDP = pectin with medium DE; HDP = pectin with high DE; DE = degree of esterification; Eq.wt = equivalent weight; MeO = methoxyl content; AUA = anhydrouronic acid.

**Table 3:** The log CFU/mL levels of *Lactobacillus casei* TISTR 390, *Lactobacillus plantarum* TISTR 877 and *Enterococcus faecium* were assessed in glucose-free MRS media with 2% supplementation of various types of pectin or PEHs during incubation at 37 °C for 0, 24, and 36 h. Statistically significant differences (*p* < 0.05) are indicated by distinct letters on bars within the same incubation period.

Supplements	*L. plantarum* TISTR 877			*L. casei* TISTR 390			*E. faecium* TISTR 1027		
	Time (h)			Time (h)			Time (h)		
	0	24	36	0	24	36	0	24	36
LDP	4.28 ± 0.05 ^a^	6.84 ± 0.03 ^e^	8.53 ± 0.07 ^d^	4.36 ± 0.12 ^bc^	6.58 ± 0.33 ^d^	7.16 ± 0.08 ^c^	4.31 ± 0.03 ^bc^	6.07 ± 0.06 ^f^	7.43 ± 0.09 ^d^
MDP	4.25 ± 0.02 ^a^	7.18 ± 0.03 ^d^	8.67 ± 0.01 ^c^	4.38 ± 0.03 ^abc^	7.36 ± 0.09 ^bc^	7.20 ± 0.17 ^cd^	4.22 ± 0.03 ^d^	6.46 ± 0.11 ^de^	7.79 ± 0.07 ^c^
HDP	4.25 ± 0.02 ^a^	7.15 ± 0.09 ^d^	8.66 ± 0.07 ^c^	4.49 ± 0.02 ^ab^	7.44 ± 0.09 ^b^	7.39 ± 0.12 ^c^	4.32 ± 0.07 ^bc^	7.47 ± 0.12 ^c^	8.39 ± 0.06 ^b^
LPEH	4.26 ± 0.05 ^a^	7.90 ± 0.03 ^c^	8.85 ± 0.03 ^b^	4.39 ± 0.02 ^abc^	6.99 ± 0.27 ^c^	8.34 ± 0.04 ^b^	4.31 ± 0.05 ^bc^	6.33 ± 0.14 ^e^	7.73 ± 0.03 ^cd^
MPEH	4.27 ± 0.01 ^a^	8.42 ± 0.02 ^a^	9.21 ± 0.07 ^a^	4.48 ± 0.00 ^ab^	7.61 ± 0.01 ^b^	8.46 ± 0.05 ^b^	4.44 ± 0.00 ^a^	7.78 ± 0.05 ^b^	9.05 ± 0.04 ^a^
HPEH	4.25 ± 0.05 ^a^	8.30 ± 0.01 ^b^	9.11 ± 0.03 ^a^	4.51 ± 0.07 ^a^	8.29 ± 0.01 ^a^	8.72 ± 0.02 ^a^	4.39 ± 0.00 ^ab^	8.17 ± 0.07 ^a^	9.16 ± 0.06 ^a^
Positive control	4.30 ± 0.02 ^a^	8.28 ± 0.01 ^b^	9.18 ± 0.01 ^a^	4.33 ± 0.06 ^c^	7.99 ± 0.04 ^a^	8.73 ± 0.16 ^a^	4.43 ± 0.05 ^a^	8.28 ± 0.02 ^a^	9.09 ± 0.12 ^a^
Negative control	4.22 ± 0.03 ^a^	7.20 ± 0.03 ^d^	8.29 ± 0.07 ^e^	4.35 ± 0.02 ^bc^	7.50 ± 0.12 ^b^	8.37 ± 0.08 ^b^	4.26 ± 0.00 ^cd^	6.64 ± 0.04 ^d^	8.35 ± 0.00 ^b^

LDP = pectin with low DE; MDP = pectin with medium DE; HDP = pectin with high DE; LPEH = pectin enzyme hydrolysate from LDP; MPEH = pectin enzyme hydrolysate from MDP; HPEH = pectin enzyme hydrolysate from HDP.

## Data Availability

The original contributions presented in the study are included in the article, further inquiries can be directed to the corresponding author.
